# Embedding mental health interventions in early childhood education systems for at-risk preschoolers: an evidence to policy realist review

**DOI:** 10.1186/2046-4053-3-84

**Published:** 2014-07-29

**Authors:** Normand J Carrey, Janet A Curran, Robin Greene, Alicia Nolan, Alan McLuckie

**Affiliations:** 1 IWK Health Centre, 5890 University Ave, Box 9700, Halifax, NS B3K 6R8, Canada; 2 Dalhousie University, 5869 University Avenue, PO Box 15000, Halifax, NS B3H 4R2, Canada

**Keywords:** Early childhood, Preschoolers, Mental health interventions, At-risk children, Realist review

## Abstract

**Background:**

Current early childhood systems of care are not geared to respond to the complex needs of preschoolers at risk for mental health problems in a timely, coordinated, multidisciplinary, and comprehensive fashion. Evidence-informed policy represents an opportunity for implementing prevention, promotion, and early intervention at the population or at-risk level. Exposure to risk factors as well as the presence of clinical disorders can derail the developmental trajectories of preschoolers, and problems may persist if left untreated. One way to address these multiple research-to-policy gaps are systematic reviews sensitive to context and knowledge user needs, such as the realist review. The realist review is an iterative process between research teams and knowledge users to build mid-level program theories in order to understand which interventions work best for whom and under what context.

**Methods/Design:**

The realist review employs five ‘iterative’ steps: (1) clarify scope, (2) search for evidence, (3) appraise primary studies and extract data, (4) synthesize the evidence, and (5) disseminate, implement, and evaluate evidence, to answer two research questions: What interventions improve mental health outcomes for preschoolers at risk for socio-emotional difficulties and under what circumstances do they work? and what are the best models of care for integrating mental health interventions within pre-existing early childhood education (ECE) services for at-risk children? Knowledge users and researchers will work together through each stage of the review starting with refining the questions through to decisions regarding program theory building, data extraction, analysis, and design of a policy dissemination plan. The initial questions will guide preliminary literature reviews, but subsequent more focused searches will be informed by knowledge users familiar with local needs and further building of explanatory program theories.

**Discussion:**

Policy makers want to know what works best for whom, but are faced with a wide and disparate intervention literature for at-risk children. Applying evidence-based standards is a good start, but the chain of implementation between research results and how to match interventions sensitive to local context are ongoing challenges.

**Trial registration:**

Prospero registration number: CRD42014007301.

## Background

Current early childhood education (ECE) systems of care (providers and decision-makers) are not geared to respond to the complex needs of preschoolers and parents at high risk for serious mental health problems in a timely, coordinated, multidisciplinary, and comprehensive fashion. As the number of children in early day care has grown, both in overall numbers and in amount of time each child spends in early care, so too has the number of children presenting in these settings with challenging socio-emotional difficulties [1]. Child care providers report that as many as 30% of their charges warrant special attention because of emotional regulation or behavioral difficulties [2]. As a result, preschool children with these difficulties experience an expulsion rate from day care three times greater than the rate for school-aged children [3,4]. The gap is even greater for children outside of the day care system whose difficulties are not identified until kindergarten, when the behaviors are already entrenched.

Who are these preschoolers and families at high risk? Can they be reliably identified so that they can receive appropriate support and intervention? In the ELDEQ study (Étude Longitudinale du Développement des Enfants du Québec), Japel and colleagues found that risk factors impacting child development included maternal depression, age and educational level of mothers, parental substance abuse, family violence, child health, temperament, poverty, and neighborhood safety [5,6]. These risk factors were identifiable as early as 5 months of age in infants and were predictive of hyperactivity, inattention, aggression, anxiety, and oppositional behavior in kindergarten. Poor mental health outcomes were dependent on the cumulative effect, i.e., the number of risk factors an infant experienced and the amount of time (years) a preschooler was exposed to the risk factors.

Advances in assessment and treatment of socio-emotional functioning in infants and preschoolers as well as longitudinal studies in child development over the last 20 to 30 years have demonstrated that psychopathology (the combination of environmental and genetic risk factors) can be reliably identified in preschoolers and that many of these problems may be enduring if not treated early [7,8]. Infants and preschoolers can suffer mental health disorders such as anxiety and aggression or can manifest attachment difficulties and post-traumatic stress disorder if raised by a parent with their own risk factors (mental illness, substance abuse, trauma) or in the context of family or intimate partner violence. Some children, struggling against many risk factors, will be thrown off of their normal developmental trajectory whereas other children, at genetic risk for psychiatric disorders, may show early manifestations of sub-syndromes or the full manifestation, with developmental variations, of psychopathology. Unfortunately, some children and their families are subjected to multiple environmental and genetic risk factors that interact/correlate (i.e., parental depression and adverse parenting) resulting in more negative outcomes.

Therefore, preschooler psychopathology must be conceptualized as the manifestation of psychiatric symptoms and/or impaired development within the context of sensitive parenting or secure/insecure attachments. Early identification and treatment become imperative to mitigate risk factors and increase protective factors, which in turn affect early brain development, caregiver/family interactions, and, ultimately, the developmental adaptations of growing children.

In order to match intervention to need while optimizing development, various public health models have been proposed, such as the universal/selective/indicated preventive approach [9]. Preventive universal interventions are intended to support healthy development for all children, whereas selective approaches target children with high lifetime or imminent risk (e.g., Head Start programs for poor families, home visitation). Indicated interventions target children already displaying minimal but detectable symptoms which may become major identifiable disorders. A fourth emerging level of intervention is for treating specific identifiable disorders in preschoolers and their families. Many new treatments are emerging and undergoing a first wave of evidential scrutiny at all levels of intervention. Some of these therapies are established treatments in older age groups that have been modified to account for developmental differences (e.g., cognitive behavior therapy for younger children) whereas other interventions have been specifically designed to address risk factors specific to the early years (attachment-based therapies to target changing parent-infant interactions or parental sensitivity). While this schema has some intuitive appeal, the field in general is still struggling to match what interventions will mitigate which risk factors in order to produce the best developmental outcomes.

Therefore, the case for intervening early and effectively is compelling; some studies have demonstrated that high-risk children and families benefit the most from high-quality intervention, delivered through either home or center-based care [10,11]. However, the evidence base is quite inconsistent and at times contradictory for comprehensive approaches because complex psychosocial interventions are quite context dependent [12,13]. Two separate reviews on US home visitation concluded that there were modest benefits at best from home visitation programs [14,15]. Cochrane reviews of home visitation involving disadvantaged mothers [16,17] and disadvantaged teenage mothers [18] found no difference in outcomes on parental or child risk factors. However, home-based interventions delivered by trained professionals with high adherence to model fidelity (such as the Nurse-Family Partnership) show better and more consistent results compared to less rigorous programs [19]. A Cochrane review for center-based care found favorable outcomes in child IQ, behavior difficulties, and school achievement [20], but the review was criticized for insufficient control of confounding home visitation interventions [21].

What factors account for these inconsistent results? Home or center-based programs are quite varied and must be flexible to meet the needs of their target populations (i.e., the context in which service delivery takes place). Therefore, some programs are effective under some conditions (poverty, immigration), for some groups of children (developmental age), and for some risk factors (parental risks—depression, substance abuse; child risks—aggression, trauma; family risks—intimate partner violence). Even the most rigorously tested programs with high fidelity (Nurse-Family Partnership) must be part of a comprehensive approach. For example, a home visitor may need to refer the child and family to a parenting program and needs to know which one is evidence based but also suits the client’s needs (Incredible Years? Triple P? Parents as Teachers?). Any intervention program should be seen as an entry point into a system of care, rather than as a single model of care. Furthermore, overarching principles guiding evidence-informed policymaking common to all early interventions are only now starting to emerge. For example, Astuto and Allen found that parental engagement was crucial for the success of any comprehensive intervention, whether it is home or center based [12]. At the higher policy level, different programs involved in early child development clash over ideological and historical differences on whether to focus on socio-emotional development or cognitive and literacy skills. National systems of care have differing priorities about the importance of the early years, affecting overall resource allocation for child welfare.

Programming decisions based strictly upon the current available evidence may be misleading or fail to recognize the multiple needs of at-risk children, parents, and communities. Since risk factors for children with mental health challenges are nonspecific and quite context dependent, this knowledge gap needs to be synthesized for policy makers involved in program design of developmentally sensitive services. Currently, no reviews, policy documents, frameworks, or models exist to help decision-makers design new or review existing programs (resources, training, staffing, and coordinated services) around mental health interventions within child welfare, early education, or public health systems of care.

Traditional systematic review methods which focus on measuring and reporting on effectiveness of interventions are not easily applied to interventions that take place in ‘real world’ health and social systems that are more complex and uncontrolled than research-based settings. Implementation science brings an evaluation framework to specific patient populations with complex needs [22]. A further refinement of implementation science specifically designed to take into account how interventions may be modified by context is the realist review [13]. This type of review requires a broader examination of all relevant information than would typically occur in a meta-analysis. It is an iterative process integrating and synthesizing knowledge from the scientific literature with input from knowledge users (KUs) familiar with local needs and barriers. KUs are actively involved with the research team to build mid-level program theories.

The realist review approach is especially suitable for complex policy questions as it attempts to illuminate the mechanisms of how interventions work under different contexts. It makes explicit the context (C), mechanisms (M) of change, and outcomes (O) (CMO configurations) in order to identify what interventions work best, for whom, and under what circumstances. This is a particularly appropriate methodology for synthesizing complex, difficult to interpret evidence from multiple settings, i.e., merging home, community, and clinic-based interventions, and where more than one change mechanism or multiple change mechanisms acting simultaneously may be operating.

This realist synthesis comes at a time when there is a critical need for integrated knowledge in the field of ECE concerning preschoolers at high risk for socio-emotional difficulties, an at-risk population and policy area heretofore neglected. Working with knowledge users responsible for ECE policy at the provincial level, this proposal provides a novel approach with a clear, practical methodology for synthesizing knowledge relevant for policy recommendations.

## Methods/Design

Our synthesis is a collaborative initiative between researchers and knowledge users across different policy, disciplinary, and organizational boundaries. The interpretative approach inherent in this review of complex early childhood interventions will benefit from the empirical perspective of researchers coupled with decision-makers and practitioners who work in the system and therefore are knowledgeable about the context. Knowledge users and researchers will work together through each stage of the review process starting with refining the research questions through to decisions regarding data extraction, data analysis, and design of a dissemination plan. The initial research questions will serve to guide preliminary literature reviews but can be modified by knowledge users in the course of the review if deemed relevant: (1) What early childhood interventions improve mental health outcomes for preschoolers at risk for socio-emotional difficulties and under what circumstances do they work? and (2) What are the best models of care for integrating mental health interventions within pre-existing ECE services for children at high risk for socio-emotional difficulties?

The primary focus of a realist synthesis is to make explicit the underlying theories about the causal mechanism of the complex intervention of interest [23]. This is accomplished by examining patterns of interaction between the context, mechanism, and outcome in a sample of primary studies. Our goal is develop a realist program theory to explain how early childhood interventions work in different contexts to achieve improved mental health outcomes for preschool children. We will employ the five steps outlined in the realist template to guide our review: (1) clarify scope, (2) search for evidence, (3) appraise primary studies, (4) extract data, (5) synthesize the evidence, and (6) disseminate, implement, and evaluate [13]. Although the steps are described in a linear fashion, progression through the steps is an iterative process and it may be necessary to revisit earlier steps as the evidence begins to emerge. More specifically, in concert with knowledge users, we will:

1. C*larify the scope of the review* by searching for candidate (middle range) theories which explain how and why early childhood interventions work under different contexts. More than one middle range theory may explain the influence of context on a mechanism to produce an outcome. Therefore, an initial scoping search will be conducted to map the range of possible relevant theories for consideration and refine the research questions. We will extract the following data from all studies included in the scoping search: study characteristics (year, population, sample size, setting, and type of intervention or program), any explicit reference to a theory underlying the intervention, and any theoretical description linking context (C), mechanism (M) of action, and outcome (O). Data will be summarized in a table, and researchers and knowledge users will work together to review the results of the mapping exercise and begin to develop a program theory. Our beginning program theory will outline the contexts in which, populations for which, and main mechanisms by which important mental health outcomes for preschool children are expected to be achieved. Researcher and knowledge user consensus around the explanatory basis of these complex programs is vital to the success of the review process, and we anticipate that this work will initially consume a large portion of our project time. We will examine the evidence from the different intervention studies and negotiate with our knowledge users to adjudicate between the rival theories and identify which theory, or set of theories, might be important for inclusion in our program theory [24]. A realist review can generate a large number of paths to explore; therefore, we will use the following questions to guide refinement of our research objectives: Do the interventions work as predicted? Which theory/theories fit best? How do the different interventions work in different settings, for different groups? What are the policy implications for the different interventions? We will keep a project journal, separate from meeting notes, to document points of contention, key decision points, and insights arising from review of the evidence and researcher-knowledge user negotiations.

2. Our purposive *search for the evidence*, focused on finding data that can be used to test our program theory, will require a combination of formal protocol-driven strategies and more informal approaches such as browsing and snowball methods (checking references of references and citation tracking). The search strategy will be guided by an information scientist and will evolve as the evidence emerges through each step of the review process. Databases to be searched include, but are not limited to, ClinicalTrials.gov; Cumulative Index to Nursing and Allied Health Literature (CINAHL); The Cochrane Library, including the Cochrane Central Register of Controlled Trials (CENTRAL); Educational Resources Information Centre (ERIC); EMBASE; Latin American and Caribbean Literature on the Health Sciences (LILACS); MEDLINE; ProQuest Nursing and Allied Health; PsycINFO; Web of Science; and WorldCat.

*Other sources*: Due to the nature of our research questions, we anticipate that gray literature searches will yield important sources of information. Gray literature sources from Canada, Europe, and the USA will be searched, including but not limited to Canadian Health Research Collection, Canadian Public Policy Collection, Grey Literature Report, and OpenSIGLE.

We will search the websites of organizations relevant to mental health and child health and the websites of federal and provincial government departments, including but not limited to Canadian Institutes of Health Research, Centre of Excellence for Early Childhood Development, CMA Infobase, Institute for Research on Public Policy, International Network for Early Child Development, International Organization for Early Intervention, National Guidelines Clearinghouse, Offord Centre for Children at Risk, World Health Organization, and World Infant Mental Health Organization. Where applicable, table of contents of specific infant mental health journals will be hand searched, e.g., *Infant Mental Health Journal*, *Child Development*, *Journal of Child Psychology and Psychiatry*, *Journal of American Academy of Child and Adolescent Psychiatry*, *Child Abuse and Neglect*, and *Developmental Psychopathology*. Key policy experts will be identified and approached as well for literature, policy, or program information.

Based on the goals of the study and the two research questions, the following *inclusion*/*exclusion criteria* will be used initially to inform the search process. After developing the CMO configurations, it is possible (consistent with a realist approach) that knowledge users and the research team may modify these criteria:

Included:

• Studies (qualitative or quantitative) focused on any type of complex intervention involving at-risk preschoolers.

• Should address risk factors or adversity in child or parent and include at least one mental health outcome.

• Should include reliable assessment of baseline mental health diagnosis or risk factors.

• Studies focused on preschoolers at risk for socio-emotional difficulties (anxiety, depression, aggression).

• Age range, 0 to 5.

• Relevant reviews and meta-analyses.

• Parenting group interventions including parents or children at high risk will be included (i.e., generic parenting groups such as groups for psychoeducation will be excluded).

Excluded:

• Studies examining autism, developmental, and communication disorders, as they are more related to child developmental-neurological factors.

• Studies involving adults over the age of 19 that do not report data for the age group 0–5 years separately.

• Studies focused on organic causes of intellectual or emotional impairment, i.e., fetal alcohol syndrome, or extreme prematurity.

• Studies focusing primarily on assessment with no intervention component, no comparison group, and no outcome measures.

Screening of studies will take place in two stages. First, two team members will independently screen all titles and abstracts for inclusion. Disagreements will be resolved by discussion or, if necessary, the senior investigator will adjudicate. In a second round of screening, the same two team members will independently examine the full text of included citations for suitability.

3. Next, we will *appraise included primary studies* using forms and tools agreed to collaboratively by the knowledge user-research team. Selection and appraisal of documents in a realist review is carried out from a heuristic perspective to enrich the proposed CMO model. Given the diverse nature of likely sources of data, we will apply a ‘fitness of purpose’ approach to appraise primary sources [25]. Two team members working independently will assess each study for relevance (Does the study add anything to our program theory?) and rigor (Are the conclusions drawn by the researcher adequately supported to make a credible contribution to test our program theory?). A summary table outlining study authors, publication date, objectives, and type of study, conclusions, and appraisal data will be developed.

4. *Data extraction* will be guided by the program theory outlined in step 1 and will evolve in an iterative manner to accommodate possible re-examination of studies as new codes emerge. One team member will conduct the first round of data extraction to examine the nature of the interventions reported in the primary sources. The basic components of a realist causal explanation include articulation of the *context*, *mechanism of action*, and *outcomes* of intervention of interest [26]. A matrix will be developed using Excel spreadsheets to organize extracted data into the basic context-mechanism-outcome configuration (step 1). In addition, we will extract any data related to relevant concepts or theories. As data are sorted, we will look for patterns (demi-regularities) to confirm or modify our program theory.

We will track how different studies are used or omitted from this first round. A second round of data extraction will follow our second search and will be used to continue to build and refine our program theory.

5. *Data analysis and synthesis* will be a joint effort of the researcher and knowledge user team and will be guided by the research questions. The primary goal of this task is refining our program theory improving our understanding of how interventions work in different contexts to achieve important mental health outcomes for preschool children. Each primary study will be examined according to how it supports, weakens, modifies, supplements, or refocuses our CMO configuration [26]. This critical approach will assist with specifying the CMO elements and developing our program theory.

6. The final step in the review process will involve reporting our findings, *development of recommendations and a plan for dissemination*. Our findings will be reported using the items listed in the RAMESES reporting standards for realist synthesis [27]. The knowledge users and researchers will work together to identify important stakeholder groups and develop recommendations taking into account the context and implementation chains of the different stakeholder groups. A brainstorming exercise will be completed using an online forum to identify potential barriers for the uptake of the recommendations. A dissemination plan will be designed to address these barriers and include successful strategies that have worked with the stakeholder groups in the past.

Integral to the success of our project’s work is the use of a knowledge broker familiar with local resources who will be an integral part of the dissemination process, merging policy, and academic perspectives. The knowledge broker will assist with crafting recommendations that are sensitive to local policy context using the appropriate lexicon.

## Discussion

Many prospective and retrospective longitudinal studies of the risk factors that can affect developmental trajectories during a period of rapid brain growth have demonstrated that this sensitive period can be a tipping point leading to life-long health or disorder [28]. Within these trajectories, certain subgroups of children are more at risk due to exposure to multiple, cumulative, and prolonged environmental or genetic risk factors, such as poor attachment, maternal depression, and poverty. Infant mental health researchers have identified another subgroup of children with the onset of psychopathology (with or without environmental triggers), highlighting an early predisposition to partial or full psychiatric disorders such as disruptive behavior or anxiety disorders. These longitudinal studies highlight that it is no longer acceptable to adopt a ‘wait and see’ attitude coupled with the prevailing attitude that ‘it is just behavior’ and ignore the evidence that preschoolers can experience intense psychological distress with potential long-term sequelae. From the treatment perspective, ‘not doing anything’ is also a missed opportunity for preventive interventions within this window of rapid development.

Policymakers want to know what works best and for whom, but are faced with a wide and disparate literature on intervention in the early years. Applying evidence-based standards to interventions is a good start, but the chain of implementation between research results and how to match interventions to local context are ongoing challenges. One way to address these multiple research-to-policy gaps is by using systematic review methodologies sensitive to local context and knowledge user needs, such as the realist review. Our first research question is formulated to address this specific need around evidence-based interventions for preschoolers at high risk for socio-emotional challenges. In addition, systemic factors such as lack of coordination between departments mandated with child development, continuity of care across developmental phases, simultaneous parallel services for children and parents (two generation models), and uneven specialized personnel training have hampered a consistent approach to intensive and continuous interventions for this population, the focus of our second research question. Working with local knowledge users, the research team will synthesize evidence and contextual factors to shape policy recommendations around embedding mental health services for at-risk preschoolers.

## Abbreviation

KU: Knowledge user.

## Competing interests

The authors declare that they have no competing interests.

## Authors’ contributions

NC and JAC designed the original study and developed the first draft of the manuscript. NC revised the protocol for journal submission. AM contributed to editing the first draft. RG and AN formatted the text for the final draft. All authors reviewed and approved the final draft.
